# Angiopoietin-1 Regulates Brain Endothelial Permeability through PTPN-2 Mediated Tyrosine Dephosphorylation of Occludin

**DOI:** 10.1371/journal.pone.0130857

**Published:** 2015-06-19

**Authors:** M. Rizwan Siddiqui, Chandra S. Mayanil, Kwang Sik Kim, Tadanori Tomita

**Affiliations:** 1 Division of Pediatric Neurosurgery, Ann & Robert H. Lurie Children’s Hospital of Chicago, Northwestern University Feinberg School of Medicine, Chicago, IL, United States of America; 2 Department of Neurological Surgery, Northwestern University Feinberg School of Medicine, Chicago, IL, United States of America; 3 Division of Pediatric Infectious Diseases, Johns Hopkins University School of Medicine, Baltimore, MD, United States of America; University of Kansas Medical Center, UNITED STATES

## Abstract

**Objective:**

Blood brain barrier (BBB) breakdown and increased endothelial permeability is a hallmark of neuro-vascular inflammation. Angiopoietin-1 (Ang-1), a Tie-2 receptor agonist ligand, is known to modulate barrier function of endothelial cells; however the molecular mechanisms related to Ang-1 mediated repair of Tight Junctions (TJs) in brain endothelium still remain elusive. In this study, we investigated a novel role of non-receptor protein tyrosine phosphatase N-2 (PTPN-2) in Ang-1 mediated stabilization of tight junction proteins.

**Method and Result:**

To study the barrier protective mechanism of Ang-1, we challenged human brain microvascular endothelial cells *in-vitro*, with a potent inflammatory mediator thrombin. By using confocal microscopy and transwell permeability assay, we show that pretreatment of brain endothelial cells with Ang-1 diminish thrombin mediated disruption of TJs and increase in endothelial permeability. We also found that Ang-1 inhibits thrombin induced tyrosine phosphorylation of Occludin and promote Occludin interaction with Zona Occludens-1 (ZO-1) to stabilize TJs. Interestingly, our study revealed that depletion of PTPN-2 by siRNAs abolishes Ang-1 ability to promote tyrosine dephosphorylation of Occludin, resulting Occludin disassociation from ZO-1 and endothelial hyperpermeability.

**Summary:**

Collectively, our findings suggest that in brain endothelial cells blocking PTPN-2 mediated tyrosine phosphorylation of Occludin is a novel mechanism to maintain BBB function, and may offer a key therapeutic strategy for neuro-inflammatory disorders associated with BBB disruption.

## Introduction

The vascular endothelial monolayer forms a semi-permeable barrier between the blood vessel and brain parenchyma called the BBB. The integrity of the BBB endothelial monolayer is largely maintained by TJ proteins which link adjacent endothelial cells (ECs) and allow them to tightly regulate paracellular transport of molecules, ions and leukocytes and thus maintain central nervous system (CNS) homeostasis [1]. Disruption of vascular integrity and increased endothelial permeability commonly occur in a variety of disease states such as stroke, trauma, tumor, infection, degenerative disorders etc., leading to the formation of cerebral edema. It is an important pathogenic and amplification mechanism in these diseases [2–5]. Furthermore, alterations of the BBB and increase in vascular permeability have been evident in septic shock patients [6]. TJ proteins of brain ECs are composed of a group of transmembrane molecules including Claudins, Occludin and JAMs [1, 7]. Occludin, the first integral TJ protein to be identified interacts with scaffolding protein ZO-1 through its C terminal domain and subsequently to the actin cytoskeleton [8, 9]. Occludin in particular is crucial in regulation for TJ barrier functions. Multiple inflammatory mediators increased permeability and inflammation by augmenting tyrosine phosphorylation of Occludin and decreasing its binding affinity to ZO-1 [10–15]. The level of tyrosine phosphorylation of proteins is regulated by protein tyrosine kinases (PTKs) and protein tyrosine phosphatase (PTPs). The role of PTPs is particularly important, by dephosphorylating Occludin, PTPs can facilitate junctions annealing and resolution of vascular hyperpermeability. The PTPN-2 (also known as T-cell protein tyrosine phosphatase), a ubiquitously expressed, non-receptor tyrosine phosphatase is crucial for inflammation regulation, as deletion of the PTPN-2 gene results in progressive systemic inflammation [16]. Additionally, reduced expression of PTPN-2 worsens IFN-γ mediated barrier dysfunction [17]. More recently, PTPN-2 has been shown to directly interact with and dephosphorylate the gap junction protein connexin-43 and stabilize gap junctions [18].

Angiopoietin-1 (Ang-1) belongs to a family of glycoproteins that serve as ligand for the ECs specific receptor tyrosine kinase (RTK) called Tie-2 receptor [19, 20]. Genetic deletion of Ang-1 is associated with defective embryonic vasculature [21]. In adult vessels, Ang-1/Tie-2 signaling maintains systemic vascular quiescence by negatively regulating endothelial hyperpermeability and inflammation. Systemic administration of Ang-1 in mice inhibits endotoxin induced vascular leakage. In ischemic rat brains, Ang-1 counteracts VEGF induced increased in BBB permeability. Ang-1 also blocks VEGF induced endothelial permeability by inhibiting Src mediated phosphorylation of VE-cadherin [22–25]. Therefore, a precise level of tyrosine phosphorylation of junctional proteins is required to maintain vascular homeostasis. Although previous studies show anti-hyperpermeability functions of Ang-1, the specific mechanisms of how Ang-1 protects against vascular leakage at the level of TJs in brain endothelial cells are not yet fully understood. Therefore, the overlapping roles of Ang-1 and PTPN-2 for maintaining proper barrier functions prompted us to hypothesize that Ang-1/Tie-2 signaling may maintain vascular integrity through PTPN-2.

To test our hypothesis, we used thrombin as an agonist to establish *in-vitro* ECs permeability assay and study Ang-1 functions to counteract deleterious effects of thrombin. We also propose to investigate whether Ang-1 requires PTPN-2 to promote the interaction of Occludin with ZO-1 and its junctional localization to stabilize brain endothelium after thrombin challenge.

## Materials and Methods

### Antibodies and chemicals

Human r-Ang-1 was purchased from R&D Systems (Minneapolis, MN). Thrombin was purchased from Enzyme Research Laboratories (South Bend, IN). Rabbit monoclonal anti-PTPN-2 antibody was from OriGene Technologies, Inc (Rockville, MD, USA). Rabbit polyclonal anti-Claudin-5, mouse monoclonal anti-PY-20 (clone 4G10) antibody was purchased from Millipore (Billerica, MA). Mouse monoclonal anti-ZO-1, rabbit polyclonal anti-Occludin antibody and DAPI were purchased from Invitrogen (Life Technologies, Grand Island, NY, USA). Rabbit polyclonal anti-Tie-2 antibody and protein A/G beads were purchased from Santa Cruz Biotechnology Inc (Dallas, TX, USA). Mouse anti-Na^+^-K^+^ ATPase β3 was from B.D. Biosciences (San Jose, CA). Rabbit anti phospho-p44/42 MAPK (Erk1/2) (Thr202/Tyr204), anti- p44/42 MAPK (Erk1/2) and anti actin was from Cell Signaling Technology (Danvers, MA). FITC conjugated secondary antibody and affinipure goat anti rabbit polyclonal light chain specific secondary antibody was from Jackson ImmunoResearch (West Grove, PA). ON-TARGET plus human PTPN-2 siRNA (ID: J-008969-05-0005) and mismatch control, transfection reagents were purchased from Dharmacon. All other chemicals were obtained from Sigma-Aldrich (St Louis, MI, USA).

### Cell culture, treatment and transfection

Human brain micro-vascular endothelial cells (HBMECs) were cultured and grown as published earlier by Stins et al. 1997 [26]. For Ang-1 and thrombin treatments, ECs were starved in RPMI-2 supplemented with 0.2% FBS for 4 hr. 70–80% confluent monolayer of ECs were transfected using siRNA transfection reagent from Dharmacon in accordance with the manufacturer’s instructions.

### Isolation of sub cellular fractions

After thrombin or Ang-1/thrombin treatment, ECs monolayer was washed with PBS, scraped and pelleted by centrifugation at 1000×*g* for 5 min. Sub cellular fractions were isolated by using Subcellular Protein Fractionation Kit for cultured cells from Thermo Scientific (Waltham, MA).

### Co-immunoprecipitation and western blot analysis

ECs were lysed in modified RIPA buffer (50 mM Tris-HCl, pH 7.5, 150 mM NaCl, 1 mM EDTA, 0.5% Sodium Deoxycholate, 1% Triton X-100, 1 mM Na_3_VO_4,_ 0.1% SDS, 10 μg/ml Aprotinin, 10 μg/ml Leupeptin and 100μM PMSF). For co-immunoprecipitation experiments, after treating ECs with agonists, 350 μg of total cellular protein was incubated with 2.5 μg of primary antibody overnight at 4°C, and precipitates were collected with 30μl of protein A/G agarose beads. Western blotting was performed as described earlier [27]. Molecular mass standards are indicated next to each blot in kilodaltons. Each immunoprecipitation assay was standardized with respect to their IgG control as shown in S1 Fig.

### *In-vitro* permeability assay

The permeability of HBMECs monolayer to Evans Blue labeled Albumin (EBA) was determined as described [27]. ECs were grown to confluence on transwell inserts with 0.4-μm pore size (Corning transwell polyester membrane cell culture inserts cat. no. CLS3460). Upper (luminal) and lower (abluminal) chambers were filled with HBSS containing 0.5% BSA and 20mM HEPES. After 30 min equilibration period, the luminal chamber was loaded with 0.057% EBA. Samples from the abluminal chamber were collected every 15 min for 120 min after 100nM of thrombin treatment. To determine the barrier protective effects of Ang-1, the endothelial monolayer was pre-incubated with 100ng/ml of Ang-1 for 15 min followed by thrombin treatment. The concentration of accumulated EBA was determined by measuring optical density at 620nm wavelength by Perkin Elmer Wallac 1420 Victor2 microplate reader. The rate of EBA clearance from the luminal chamber was determined by least-squares linear regression between 15 and 120 min.

### Immunofluorescence analysis

Cell fixation and staining was performed as described [27]. After activation with different agonists, the ECs monolayer was fixed with 4% paraformaldehyde and permeabilized with 0.2% Triton X-100. Monolayer was blocked in 1% BSA and incubated with rabbit polyclonal anti- Occludin (Invitrogen, 1:100). FITC-labeled secondary antibody (dilution 1:100) (Jackson ImmunoResearch) was used for the detection. Images were obtained by using a confocal microscope (Zeiss LSM 510 META) equipped with 63X, 1.2 NA oil immersion objective and an Ar ion and dual HeNe lasers. Numbers of gaps between ECs was counted by using Image-J software. Adobe Photoshop was used to assemble images. Scar bars 10μm.

### Statistical analysis

Significance between different experiments was examined by a two-tailed Student's *t* test. Differences in mean values were considered significant at P<0.05. All experiments were conducted in triplicate.

## Results

### Ang-1 prevents disruption of inter-endothelial junctions and endothelial hyperpermeability induced by thrombin

**Fig 1:**
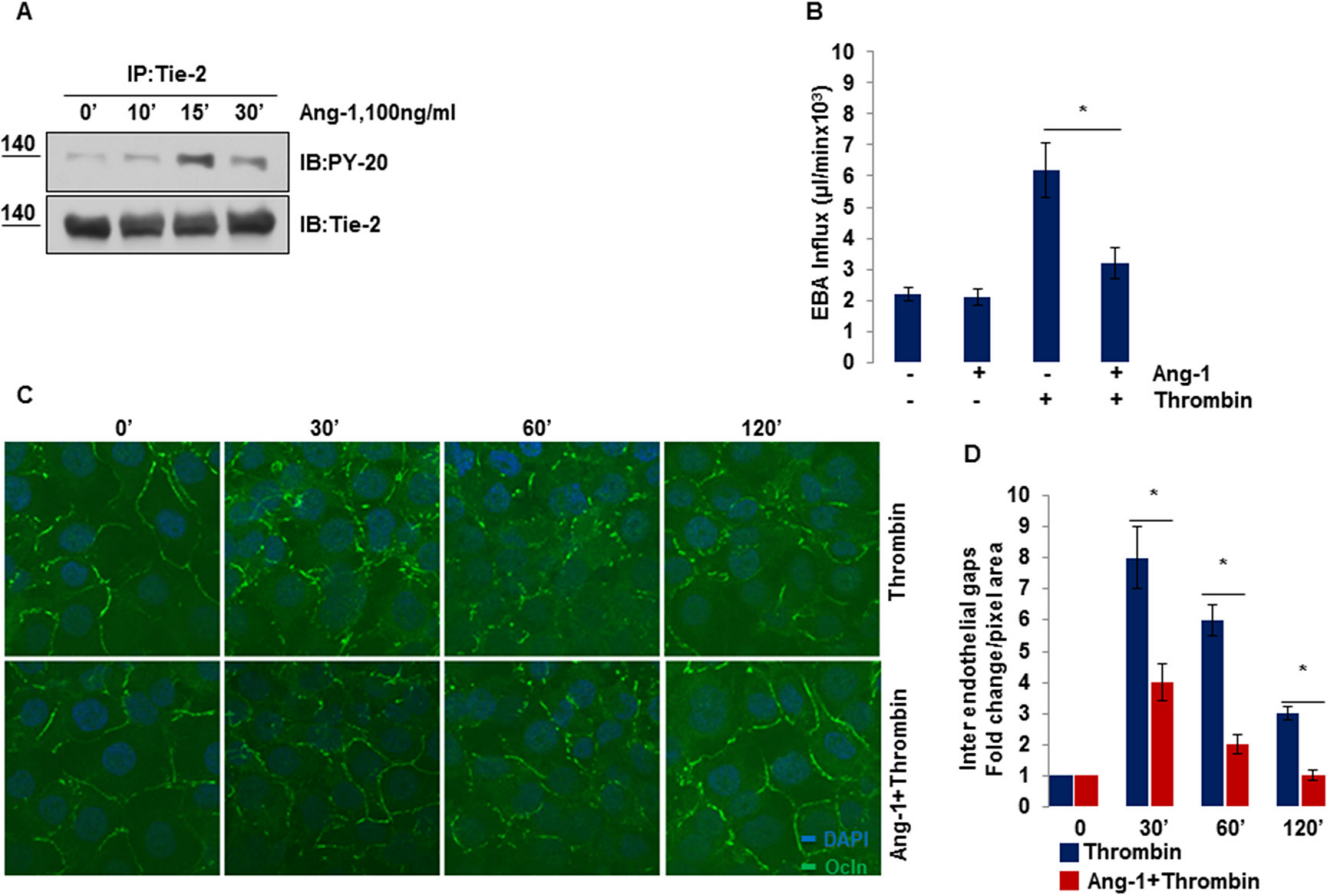
Ang-1 prevents disruption of inter-endothelial junctions and endothelial hyper-permeability induced by thrombin. (**A)** Serum starved HBMECs were stimulated with 100ng/ml of Ang-1 for different times. Tyrosine phosphorylation level of Tie-2 was determined by immunoprecipitating Tie-2 followed by Western blotting for PY-20 antibody. (**B)** Transendothelial EBA tracer permeability was determined in serum starved HBMECs unstimulated or stimulated with Ang-1 (100ng/ml for 15 min) or with thrombin (100nM for 120 min) or incubated with Ang-1 followed by thrombin treatment. Bar graphs show the average ±SEM, *P<0.05. (**C)** HBMECs were treated with 100nM thrombin alone or after Ang-1 (100ng/ml for 15 min) for indicated times. Cells were fixed, permeabilized and stained for Occludin. Immunofluorescent staining and confocal microscopy was performed as described in Methods. Scale bar = 10μM. **(D)** Quantification of number of inter-endothelial gap (shown as fold change quantified using Occludin staining described in (**C**), *P<0.05.

We first assessed Ang-1 mediated Tie-2 receptor activation as a function of time. Confluent HBMECs monolayers were treated with Ang-1 (100ng/ml) for varying durations. As described in (Fig 1A), Ang-1 induced rapid tyrosine phosphorylation of Tie-2 receptor within 15 min, which lasted until the 30 min time period; therefore, in the subsequent experiments we used the 15 min time point to study Ang-1 signaling. To determine the protective effects of Ang-1 on endothelial barrier, we treated HBMECs with a potent stimulus thrombin alone (known to markedly disrupt endothelial barrier and increase paracellular permeability [27] or pre-incubated with Ang-1 before thrombin treatment, and studied the passage of EBA tracers through endothelial monolayer as a measure of paracellular permeability. Our results show (Fig 1B), that thrombin prominently enhanced trans-endothelial EBA flux whereas pretreatment with Ang-1 significantly blocked thrombin induced increase in endothelial permeability.

We also determined the barrier protective effects of Ang-1 by immunostaining of ECs with the tight junction protein; Occludin. Confluent monolayers of ECs were challenged with thrombin alone or pretreated with Ang-1 and then challenged with thrombin for different times. Cells were fixed and stained with Occludin to visualize the integrity of TJs. Our immunocytochemistry results show (Fig 1C) reduced expression of Occludin at junctions, maximum gap formation and discontinuity observed at 30 min of thrombin challenge before it began to decrease. In comparison, pretreatment of ECs with Ang-1 markedly prevented thrombin induced barrier disruption and significantly sustained the Occludin protein at TJs. Quantification of the number of gap formations is shown in (Fig 1D). Thus, data from the EBA flux assay and fluorescent imaging show that Ang-1 protected against thrombin induced increase in brain endothelium permeability.

### Ang-1 inhibits thrombin induced disassociation of Occludin with ZO-1 and its membrane dis-localization

**Fig 2:**
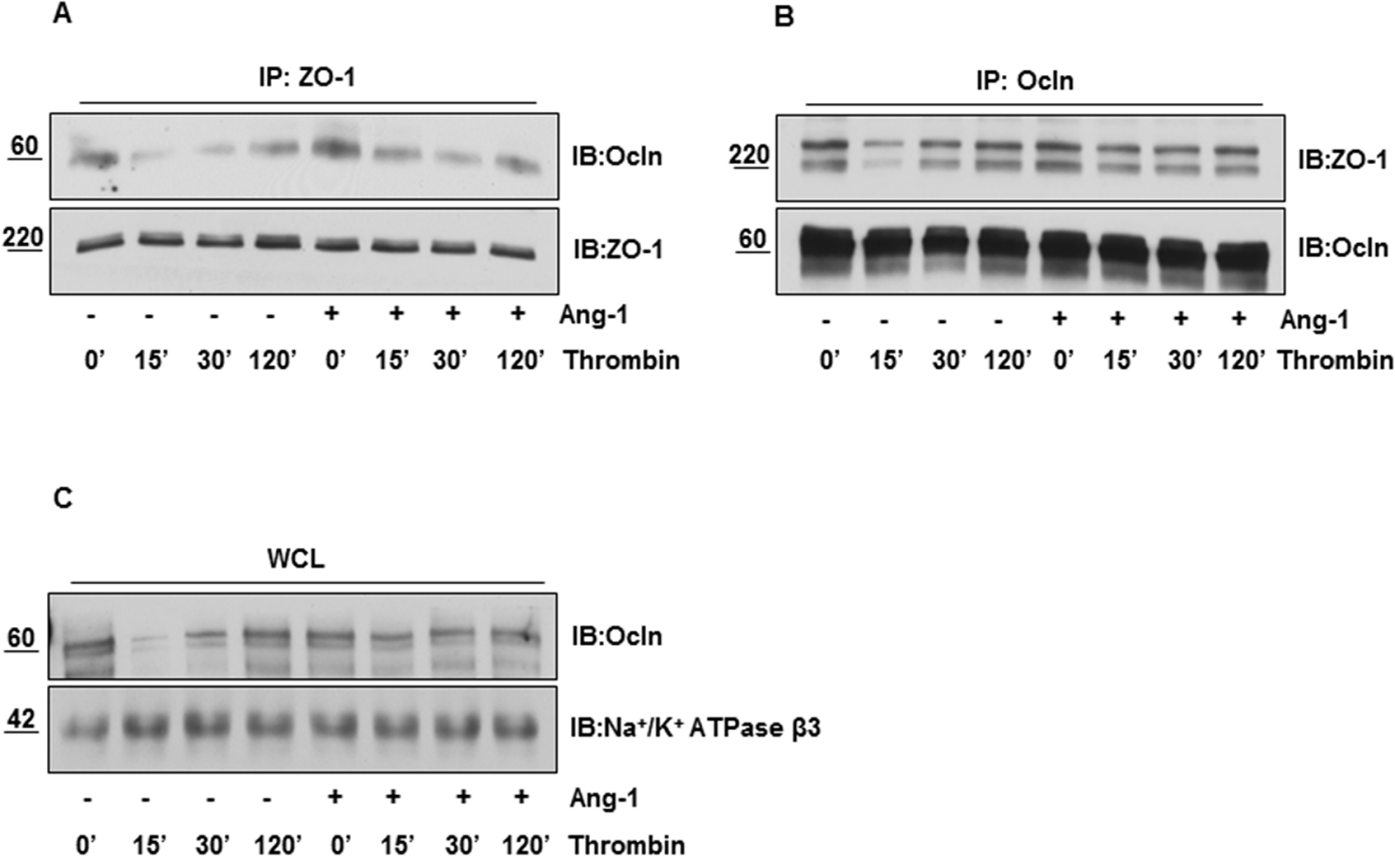
Ang-1 inhibits thrombin induced disassociation of Occludin with ZO-1 and its membrane dis-localization. Serum starved HBMECs were treated with 100nM thrombin alone or after Ang-1 (100ng/ml for 15 min) for indicated times. Cell lysates were immunoprecipitated with **(A)** anti-ZO-1 antibody and probed with anti-Occludin antibody, **(B)** anti-Occludin antibody and probed with anti-ZO-1 antibody. **(C)** After treating HBMECs with thrombin alone or pretreated with Ang-1 (100ng/ml for 15 min) followed by 100nM thrombin challenged for different times, plasma membrane fractions were prepared, separated on SDS gel and probed for Occludin. Na^+^-K^+^ ATPase β3 was used as a membrane marker.

Since endothelial barrier integrity depends on Occludin’s interaction with ZO-1 and localization of Occludin in membrane, we immunoprecipitated ZO-1 and Occludin from HBMEC lysates and immunoblotted with Occludin and ZO-1 respectively. Co-immunoprecipitation experiments show that ZO-1/Occludin interacted in control HBMECs, but thrombin treatment significantly impaired this interaction in a time dependent manner. Interestingly, pre-treatment of ECs with Ang-1 markedly mitigated thrombin induced disruption of ZO-1 and Occludin (Fig 2A and 2B). Next, we analyzed whether thrombin stimulation altered Occludin localization at the membrane. Immunoblot analysis with membrane fractions show that thrombin treatment decreased Occludin distribution in membrane fraction in time a dependent manner. Furthermore, subcellular localization of Occludin in the membrane was sustained if brain ECs were pre-treated with Ang-1 before thrombin challenge (Fig 2C), suggesting that Ang-1 may regulate TJ integrity by promoting ZO-1/Occludin interaction and preventing Occludin redistribution from the plasma membrane.

### Ang-1 diminishes thrombin mediated tyrosine phosphorylation of Occludin

**Fig 3:**
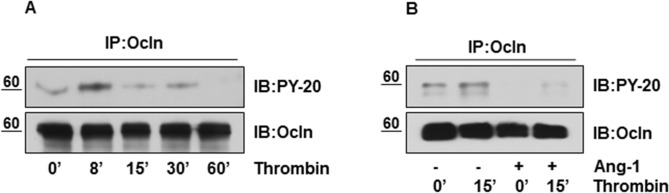
Ang-1 diminishes thrombin mediated tyrosine phosphorylation of Occludin. (**A)** Serum starved HBMECs were treated with 100nM thrombin for indicated time periods. Cellular proteins were immunoprecipitated with anti-Occludin antibody followed by immunoblotting using anti-PY-20 antibody. Blot was reprobed with anti-Occludin antibody. (**B)** ECs were stimulated with thrombin (100nM for 15 min) or pretreated with 100ng/ml Ang-1 for 15 min followed by thrombin challenge (100nM for 15 min). Cell lysates were immunoprecipitated with anti-Occludin antibody and probed with PY-20 antibody to detect tyrosine phosphorylation of Occludin. The same blot was reprobed for total Occludin.

Phosphorylation of the Occludin protein at tyrosine residues has been linked to the destabilization of the TJ complex and increased TJ permeability [12], and hence may contribute to the barrier annealing effect of Ang-1. We sought to determine the effects of thrombin on tyrosine phosphorylation of Occludin. HBMECs were treated with thrombin for different time periods and cell lysates were immunoprecipitated with anti-Occludin antibody and probed with anti-phosphotyrosine antibody. Western blot analysis reveals rapid tyrosine phosphorylation of Occludin induced by thrombin with respect to time (Fig 3A). By contrast, pre-treatment of HBMECs with Ang-1 followed by 15 min thrombin challenge markedly mitigated thrombin induced tyrosine phosphorylation of Occludin (Fig 3B). Hence, our findings suggest that Ang-1 may regulate TJ integrity by interfering with thrombin’s ability to promote tyrosine phosphorylation of Occludin.

### Deletion of PTPN-2 abolishes barrier protective function of Ang-1

**Fig 4:**
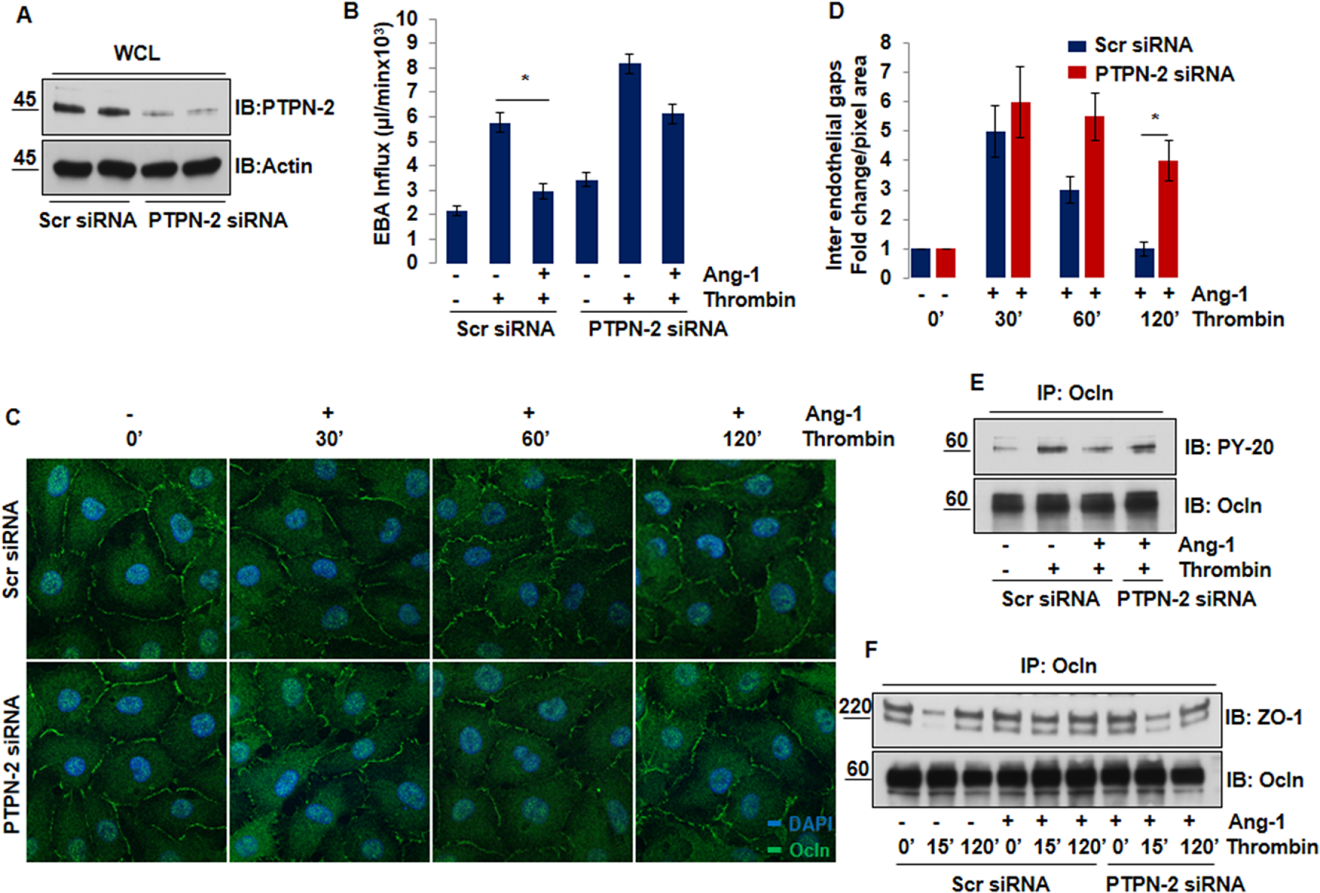
Deletion of PTPN-2 abolishes barrier protective function of Ang-1. **(A)** HBMECs were transfected with 50nM of either scramble or PTPN-2 siRNA. After 48 hours of transfection, cell lysate was collected, and immunoblotted with anti-PTPN-2 antibody to see the knockdown efficiency. The same blot was reprobed for Actin. (**B)** EBA permeability was determined in HBMECs monolayers transfected by 50nM scramble siRNA or PTPN-2 siRNA. Cells were treated with 100nM of thrombin for 120 min alone and after Ang-1 (100ng/ml for 15 min). Bar graphs show the average ±SEM, *P<0.05. (**C)** Representative confocal images of Occludin staining to show cell-cell contact in confluent HBMECs transfected with either scramble siRNA or PTPN-2 siRNA. ECs were then pretreated with Ang-1 (100ng/ml for 15 min) followed by thrombin treatment. Scale bar = 10μM. (**D)** Quantification of number of inter-endothelial gaps is shown in fold change. Data are represented as mean ±SEM *P<0.05. (**E)** HBMECs were transfected with 50nM scramble siRNA or PTPN-2 siRNA for 48 hr. Serum starved ECs monolayer was stimulated by thrombin (100nM for 15 min) alone and after Ang-1 pretreatment (100ng/ml for 15 min). Level of pY-Occludin and total Occludin was determined by immunoprecipitation and Western blot analysis. (**F)** ECs were transfected with 50nM of scramble or PTPN-2 siRNA for 48 hr followed by exposure to 100nM of thrombin in absence or presence of Ang-1 pretreatment (100ng/ml for 15 min). Immunoprecipitation was used to study Occludin/ZO-1 interaction.

We next addressed the role the tyrosine phosphatase PTPN-2 as a mediator of Ang-1 induced tyrosine dephosphorylation of Occludin. In order to test this hypothesis, HBMECs were transfected with PTPN-2 or Scramble (Scr) siRNA. Knockdown of PTPN-2 by siRNA was confirmed by Western blot analysis indicated in (Fig 4A). Scr and PTPN-2 siRNA transfected ECs were treated with either thrombin or pre-incubated with Ang-1 followed by thrombin challenge. Endothelial permeability was studied by trans-well permeability assay. Pretreatment of Ang-1 significantly inhibits thrombin induced increase in junctional permeability in Scr-siRNA transfected ECs. Interestingly, PTPN-2 silencing abolished the barrier protective effects of Ang-1 and Ang-1 failed to decrease thrombin induced endothelial hyperpermeability. However, knockdown of the PTPN-2 gene per se did not significantly alter basal endothelial barrier permeability as compared to scramble siRNA (Fig 4B).

We also determined the role of PTPN-2 in mediating the anti-hyperpermeability effects of Ang-1 using confocal microscopy as shown in (Fig 4C). HBMECs were transfected with scramble siRNA or PTPN-2 siRNA, pre-incubated with Ang-1 for 15 min before thrombin treatment for different time points. Cells were fixed and stained with anti-Occludin antibody to visualize TJs. Pretreatment of ECs with Ang-1 significantly attenuated thrombin induced barrier disruption in scramble siRNA transfected ECs (upper panel). In comparison, knockdown of PTPN-2 remarkably reduced Ang-1 ability to preserve Occludin at TJ and re-seal inter-endothelial gaps formed by thrombin (lower panel), (Fig 4C). Quantification of inter-endothelial gaps is shown in (Fig 4D).

Based on these results, we further investigated whether PTPN-2 could play a role in Ang-1 mediated tyrosine dephosphorylation of Occludin and its interaction with ZO-1. Scr and PTPN-2 siRNA transfected HBMECs were treated with thrombin for 15 min or pre-incubated with Ang-1 followed by 15 min of thrombin challenge; lysates were immunoprecipitated with anti-Occludin antibody and blotted with anti-phosphotyrosine antibody. Transfection of siRNA against the PTPN-2 gene markedly dampened Ang-1 ability to prevent thrombin induced tyrosine phosphorylation of Occludin (Fig 4E). To study the effect of PTPN-2 silencing on maintaining Occludin/ZO-1 interaction, Scr and PTPN-2 siRNA transfected ECs were treated with thrombin alone or pretreated with Ang-1 followed by thrombin challenge as indicated in Fig 4F. Lysates were immunoprecipitated with anti-Occludin antibody and blotted for ZO-1. Notably, Ang-1 partly restored Occludin/ZO-1 interaction in PTPN-2 depleted brain ECs (Fig 4F), suggesting that Ang-1 requires PTPN-2 to inhibit tyrosine phosphorylation of Occludin and its association with ZO-1 to antagonize thrombin mediated TJ disruption and increase endothelial permeability.

### PTPN-2 depletion has no effect on Ang-1 mediated Tie-2 and Erk activation, and on expression of TJ proteins

**Fig 5:**
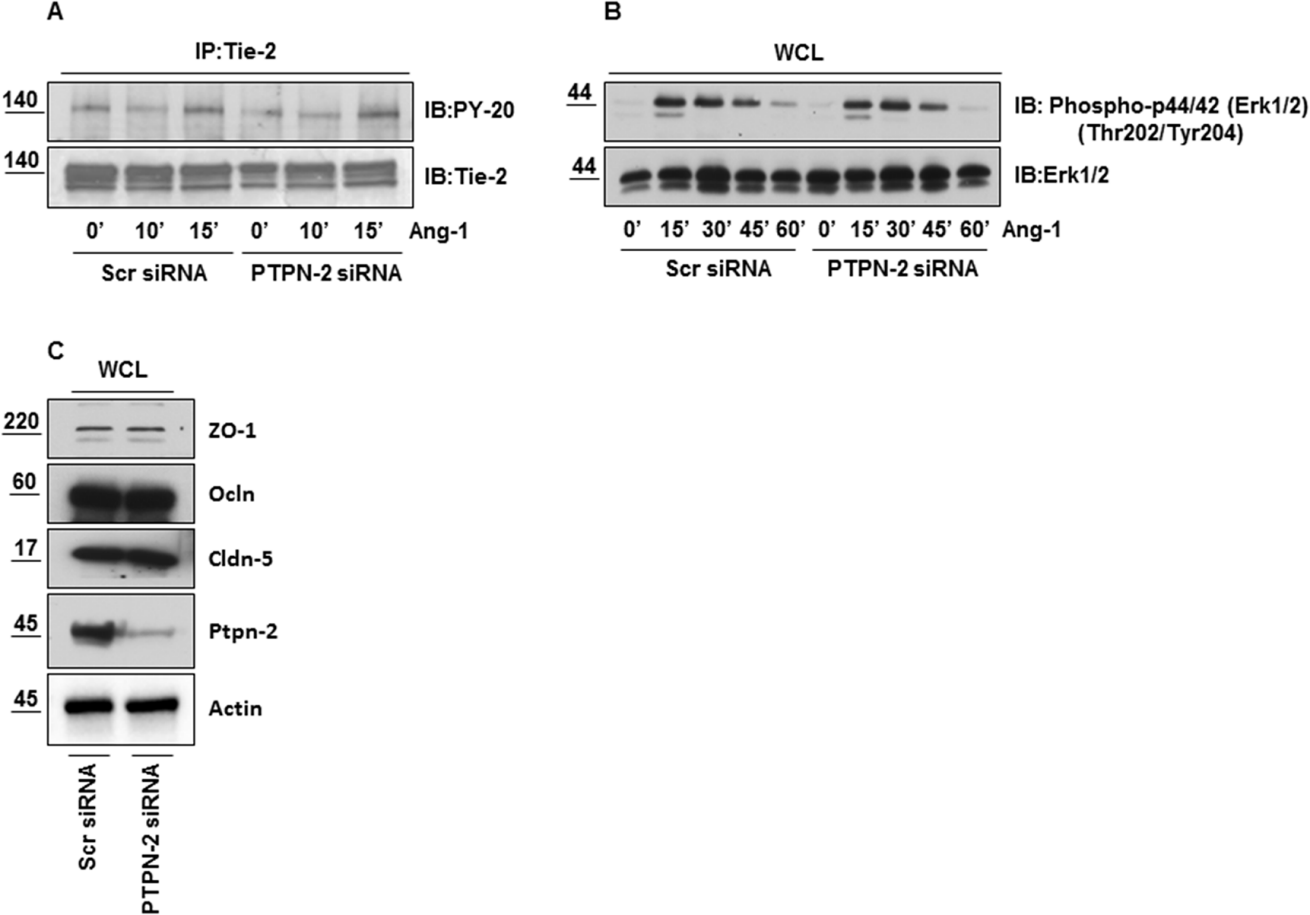
PTPN-2 depletion has no effect on Ang-1 mediated Tie-2 and Erk activation, and on expression of TJ proteins. Scramble and PTPN-2 siRNA transfected HBMECs were activated with 100ng/ml of Ang-1 for indicated times. (**A)** Immunoprecipitation of Tie-2 protein followed by immunoblotting with anti-PY-20 antibody; blot was reprobed with anti-Tie-2 antibody. (**B)** Whole cell lysates were immunoblotted with anti-Phospho-p44/42 (Erk1/2) (Thr202/Tyr204) and anti-Erk1/2 antibody. (**C)** Whole cell lysates were analyzed by Western blotting to study the expression of major TJ proteins with anti-ZO-1, anti-Occludin, anti-Claudin-5 and anti-PTPN-2 antibody. Actin used for loading control.

The tyrosine phosphatases can themselves bind and modulate activation of RTK. In endothelial cells PTPN-2 directly interacts with and dephosphorylates VEGFR-2 and inhibits its kinase activity [28]. Therefore, it was important to determine whether knockdown of PTPN-2 could alter Ang-1 mediated Tie-2 receptor activation and its downstream signaling. Scramble and PTPN-2 siRNA transfected HBMECs were challenged with Ang-1 for varying durations. Cell lysates were immunoprecipitated with anti-Tie-2 antibody and immunoblotted with anti-phosphotyrosine antibody. We observed no difference in Ang-1 mediated tyrosine phosphorylation of Tie-2 between Scr and PTPN-2 siRNA transfected ECs as demonstrated in (Fig 5A). Knockdown of PTPN-2 also had no effect on ERK activation either basally or after Ang-1 treatment (Fig 5B). These results suggest that PTPN-2 does not directly modulate Ang-1/Tie-2 signaling. We also determined expression levels of the TJ proteins ZO-1, Occludin and Claudin-5 after PTPN-2 deletion since any alterations in their expression could also explain why Ang-1 failed to prevent the thrombin induced hyperpermeability in the PTPN-2 depleted ECs; however we observed no significant difference in the baseline expression of TJ proteins in PTPN-2 depleted HBMECs compared to their control ECs (Fig 5C).

## Discussion

Dysfunctional BBB and persistent increase in vascular permeability is one of the primary causes of excessive inflammation in neurological diseases such as cerebral edema, stroke, traumatic brain injury and multiple sclerosis. Therefore, developing therapies to counter vascular hyperpermeability related disease conditions is urgently needed. Previous studies have demonstrated a potential role of thrombin in the development of brain edema and neuro-inflammation [29–31]. Thus, using thrombin as a canonical inflammatory mediator to induce barrier dysfunction in HBMECs is a useful model to understand the molecular mechanisms for BBB regulation. Earlier studies have defined the functions of Ang-1 in maintaining vascular quiescence by inhibiting increased vascular permeability against inflammatory mediator [25]; however, the mechanisms by which the Ang-1/Tie-2 axis orchestrates the signaling pathways leading to TJ stabilization in brain ECs are unknown. In this study we show that Ang-1 protects BBB functions during inflammation by enhancing brain endothelial cell to cell contacts thereby inhibiting ECs permeability. Our data demonstrate that Ang-1 prevents thrombin induced tyrosine phosphorylation of Occludin, thus resulting in stabilized Occludin/ZO-1 association at junctions. Importantly, genetic depletion of PTPN-2 abolished barrier protective effects of Ang-1 in HBMECs, thus indicating the functional importance of the Ang-1/Tie-2/PTPN-2 axis for vascular barrier integrity.

Occludin is a major component of TJs which enables the BBB to function as a restricted barrier. Localization of Occludin at the cell-cell junction with ZO-1 is important for TJ stability, as direct interaction of Occludin and ZO-1 regulates paracellular permeability [32]. Previous studies demonstrated that tyrosine phosphorylation of Occludin interferes with its association with ZO-1 and thus impairs barrier function in response to different permeability enhancing agents [11, 12]. Our study now demonstrates that increased brain ECs permeability is associated with a rapid increase in tyrosine phosphorylation of Occludin and a subsequent reduction in Occludin content at TJs in response to thrombin. In line with this observation, tyrosine phosphorylation of Occludin is also involved in the progression of cerebral ischemia [33]. Another recent study showed that VEGF induced blood retinal barrier disruption required Occludin Serine phosphorylation [34]. Taken together these data suggest that post-translational modifications of Occludin significantly contribute to alterations in TJ organization and vascular permeability underlining the importance of the tyrosine dephosphorylation pathway for endogenous protective response against inflammation. Here, we provide evidence that Ang-1 mediated protection of TJs in brain ECs is dependent on tyrosine dephosphorylation of Occludin.

An earlier study addressed the putative function of PTPN-2 for TJs barrier functions [35]. It showed that spermidine, a polyamine, required PTPN-2 to protect the intestinal epithelial barrier against IFN-γ; however this study failed to address the exact mechanism at the level of junctions by which PTPN-2 mediates barrier protection. We observed that in brain ECs, PTPN-2 regulates tyrosine dephosphorylation of Occludin in response to Ang-1, and depletion of PTPN-2 favored tyrosine phosphorylated Occludin and destabilization of TJs. As impaired PTPN-2 function prevents Ang-1 mediated endothelial barrier stabilization, it is tempting to speculate that the level of PTPN-2 phosphatase activity at the level of TJs determines whether vascular leakiness is abated or persistent during inflammation. An interesting quandary presented by the data is that PTPN-2 deletion did not significantly alter baseline permeability of brain ECs. This finding points to the role of PTPN-2 as a TJ regulatory phosphatase only when Occludin is phosphorylated by a permeability increasing mediator.

Endothelial specific PTPs are represented as negative regulators of RTK signaling. Blocking Tie-2 associated VE-PTP, another PTP found in the junctional complex proteins, with anti VE-PTP specific antibody stimulates tyrosine phosphorylation of Tie-2 [36], whereas activation of PTPN-2 results in inhibition of VEGFR-2 signaling in ECs [28].Therefore, it is reasonable to hypothesize that PTPN-2 might also regulate Tie-2 signaling. However, we showed that deletion of PTPN-2 does not alter Ang-1 mediated Tie-2 phosphorylation and Erk activation in brain ECs. Thus, unlike VEGFR-2, PTPN-2 might regulate the Tie-2 signaling pathway via interaction with downstream molecules. Therefore, PTPN-2 appears to have distinct actions on VEGFR-2 and Tie-2 receptor, and likely different functions in regulating AJ stability. In addition, genetic deletion of another non-receptor tyrosine phosphatase, Shp-2, enhanced permeability by dampening the expression of tight junction proteins in colon epithelial cells [37]. In contrast, PTPN-2 depletion did not modulate the expression of TJ proteins in our study. Thus we determined that perturbed barrier protective effects of Ang-1 in PTPN-2 deficient brain ECs is because of post-translational modification of Occludin rather than change in the expression level of TJ proteins.

Collectively, the Ang-1/Tie-2/PTPN-2 pathway maintains Occludin in a dephosphorylated state and allows it to prevent BBB leakage. In this regard, a complete delineation of the specific molecular mechanisms by which each class of vascular leakage factors acts may facilitate the identification of additional targets for the anti-permeability action of Ang-1, and could lead to the development of therapeutic strategies to prevent leaky vessel associated neuro-inflammatory disorders.

## Conclusions

Our study indicates that Ang-1 counteracts thrombin mediated increased in brain endothelial permeability by promoting Occludin and ZO-1 interaction. At, molecular level, Ang-1 prevents thrombin induced tyrosine phosphorylation of Occludin in a PTPN-2 dependent manner that interferes with thrombin’s ability to destabilize inter endothelial junctions, thus restoring TJs integrity in brain endothelium.

## Supporting Information

S1 Fig IgG controls for immunoprecipitation assay. Endogenous protein was precipitated by their respective antibody and normal IgG was used as a negative control in immunoprecipitation assay. **(A)** After immunoprecipitation with anti-Tie-2 antibody, endogenous Tie-2 (molecular weight ~140 kDa) was detectable by using anti-Tie-2 antibody followed by HRP conjugated secondary antibody in Western blot analysis, but not in IgG control. **(B)** After immunoprecipitation with anti-ZO-1 antibody, endogenous ZO-1 (molecular weight ~220 kDa) was detectable by using anti-ZO-1 antibody followed by HRP conjugated secondary antibody in Western blot, but not in IgG control. **(C)** After immunoprecipitation with anti-Occludin antibody, endogenous Occludin (molecular weight ~60 kDa) was detectable by using anti-Occludin antibody followed by affinipure goat anti rabbit polyclonal light chain specific secondary antibody in Western blot, but not in IgG control group.
(TIF)
