# Supplementary figures and images for: Angiopoietin-1 Regulates Brain Endothelial Permeability through PTPN-2 Mediated Tyrosine Dephosphorylation of Occludin
Source: PLoS One. 2015 Jun 19;10(6):e0130857. doi: 10.1371/journal.pone.0130857 (PMC4474807; doi:10.1371/journal.pone.0130857)

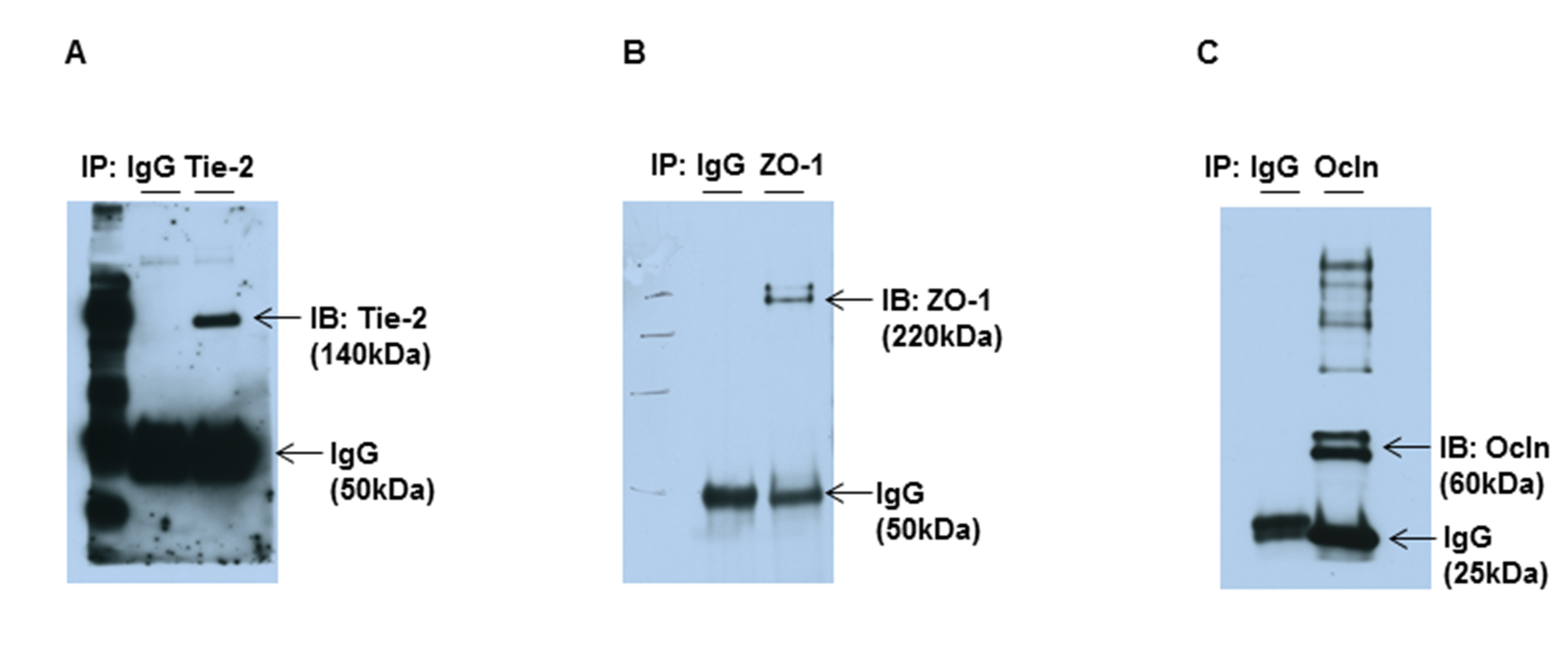

Supplement: S1 Fig — Endogenous protein was precipitated by their respective antibody and normal IgG was used as a negative control in immunoprecipitation assay. (A) After immunoprecipitation with anti-Tie-2 antibody, endogenous Tie-2 (molecular weight ~140 kDa) was detectable by using anti-Tie-2 antibody followed by HRP conjugated secondary antibody in Western blot analysis, but not in IgG control. (B) After immunoprecipitation with anti-ZO-1 antibody, endogenous ZO-1 (molecular weight ~220 kDa) was detectable by using anti-ZO-1 antibody followed by HRP conjugated secondary antibody in Western blot, but not in IgG control. (C) After immunoprecipitation with anti-Occludin antibody, endogenous Occludin (molecular weight ~60 kDa) was detectable by using anti-Occludin antibody followed by affinipure goat anti rabbit polyclonal light chain specific secondary antibody in Western blot, but not in IgG control group. (TIF) [file pone.0130857.s001.tif]
